# A Case of Postoperative Tuberculous Spondylitis with a Bizarre Course

**DOI:** 10.4055/cios.2009.1.1.58

**Published:** 2009-02-06

**Authors:** Do Whan Jeon, Bong-Soon Chang, Ul Oh Jeung, Seuk Jae Lee, Choon-Ki Lee, Min-Seok Kim, Woo-Dong Nam

**Affiliations:** Department of Orthopedic Surgery, Seoul National University College of Medicine, Seoul, Korea.; * Department of Orthopedic Surgery, Kangwon National University College of Medicine, Chuncheon, Korea.

**Keywords:** Spine, Postoperative infection, Tuberculous spondylitis

## Abstract

Postoperative infections following spine surgery are usually attributable to bacterial organisms. *Staphylococcus aureus* is known to be the most common single pathogen leading to this infection, and the number of infections caused by methicillin-resistant *Staphylococcus aureus* is increasing. However, there is a paucity of literature addressing postoperative infection with *Mycobacterium tuberculosis*. We encountered a case of tuberculous spondylitis after spine surgery. A man had fever with low back pain three weeks after posterior interbody fusion with instrumentation for a herniated intervertebral disc at the L4-L5 level. He had been treated with antibiotics for an extended period of time under the impression that he had a bacterial infection, but his symptoms and laboratory data had not improved. Polymerase chain reaction for *Mycobacterium tuberculosis* turned out to be positive. The patient's symptoms finally improved when he was treated with antituberculosis medication.

Most postoperative infections following spine surgery has occurred by bacterial organisms. *Staphylococcus aureus* is known to be the most common single pathogen, and the earlier postoperative infection is related with methicillin-resistant *Staphylococcus aureus* (MRSA) or Gram negative bacteria. However, there is little report of postoperative infection with *Mycobacterium tuberculosis*.^1^,2) We encoun-tered a case with a bizarre course of infection after spine surgery. A man had fever with low back pain three weeks after spine surgery. He had been treated with antibiotics for an extended period of time under the impression of a bacterial infection without any clinical improvement. Finally polymerase chain reaction (PCR) for *Mycobacterium tuberculosis* turned out to be positive, so he was successfully treated with antituberculosis medication.

## CASE REPORT

**Fig. 1:**
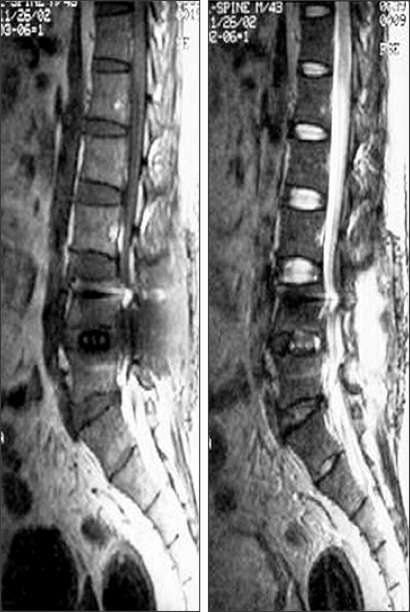
Sagittal T1- and T2-weighted images at 4 weeks post-operative. No definite sign of infection was identified.

**Fig. 2:**
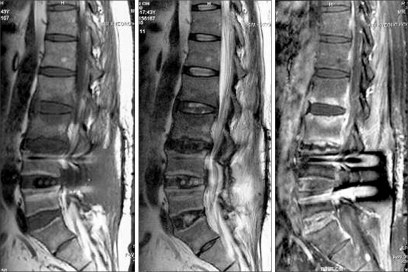
Sagittal T1- and T2-weighted and contrast-enhanced images at 6 months postoperative. Infectious spondylodiscitis was apparent at L2-S1, and anterior epidural abscess formation was apparent at the L2-L3 level.

**Fig. 3:**
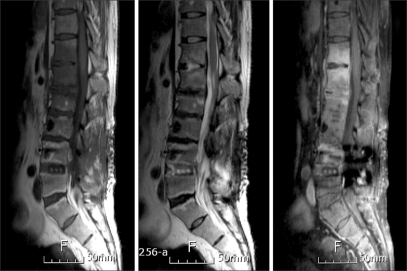
Sagittal T1- and T2-weighted and contrast-enhanced images at 13 months postoperative. MRI showed subligamentous extension of inflammation to the T12-L1 intervertebral disc.

A 44-year-old man, who had a history of complete recovery from pulmonary tuberculosis 20 years previously, underwent posterior interbody fusion with instrumentation for a herniated intervertebral disc at the L4-L5 level in October 2002 at a local neurosurgical clinic. He was symptom-free immediately after surgery, but he developed fever and low back pain at three weeks postoperative. He was treated with antibiotics under the assumption that he had a bacterial infection. No tissue cultures were taken, and no definite signs of inflammation were detected on the MRI scan performed four weeks after surgery (Fig. 1). Approximately three months of antibiotic therapy did not result in improvement of his condition, and he was referred to the department of neurosurgery at one university hospital. At the time of referral, his erythrocyte sedimentation rate (ESR) was as high as 40 mm/hr, and his C-reactive protein (CRP) was 4.25 mg/dl. Nevertheless, because no definite signs of infection were noted on MRI, he received antibiotic therapy for two more weeks. He continued to complain of persistent low back pain, and he was sent to the department of anesthesiology and pain medicine at five months postoperative to be treated with a facet joint block and sacroiliac joint block. A vertebroplasty was also performed at the L3-L5 levels, even in the absence of actual signs of compression fracture. Still, pain relief was not achieved, and ESR and CRP continued to be elevated. At six months after the initial surgery, MRI revealed signs of infectious spondylodiscitis at the L2-S1 levels and anterior epidural abscess formation at the L2-L3 level (Fig. 2). Culture of needle biopsy specimens taken from the L3-L4 and L4-L5 intervertebral discs did not lead to identification of the causative micro-organism. After two months of antibiotic therapy with cefazedone 1 g b.i.d. and gentamicin 80 mg q.d., the patient's ESR fell to 47, his CRP fell to 0.49, and his pain was relieved. When the antibiotic regimen was changed to ciprofloxacin 250 mg b.i.d., even better pain relief was achieved, and ESR and CRP dropped further to 32 and 0.34, respectively. However, discontinuation of antibiotic therapy following these improvements resulted in a relapse of low back pain, with CRP levels in normal range, in three months. An MRI obtained at that time showed subligamentous extension of inflammation to the T12-L1 intervertebral disc (Fig. 3).

**Fig. 4:**
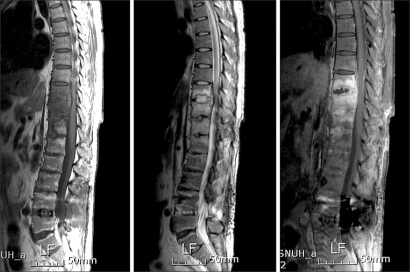
Sagittal T1- and T2-weighted and contrast-enhanced images at 22 months postoperative. MRI revealed progression of the bone marrow signal change, with enhancement of the lesion at T10-L1 and increased size of the anterior epidural abscess at T10-T12.

The patient was referred to the department of orthopedics at another university hospital, when he presented with an ESR of 37 and a CRP of 12. The patient was treated with vancomycin 1 g b.i.d. and ciprofloxacin 250 mg b.i.d. for approximately 3 months, but ESR and CRP did not improve. MRI showed extension of inflammation to the T11-T12 level. A needle biopsy of the bone was performed, and culture of the biopsy specimens was positive for *E. coli*, *Enterococcus faecalis*, and *Enterococcus faecium*, while PCR was negative for *Mycobacterium tuberculosis*. The antibiotic regimen was modified accordingly to ciprofloxacin and amikacin for 10 days. Then, because ESR and CRP were as high as 45 and 12, respectively, vancomycin and ciprofloxacin were administered instead. In short, the 6 months of antibiotic therapy administered at that hospital was ineffective in terms of pain relief. Furthermore, the in flammation spread to T10 (Fig. 4).

**Fig. 5:**
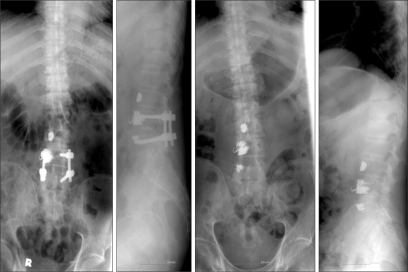
Preoperative and postoperative X-rays for the second operation. After posterior instruments were removed, we performed curettage and grafting with autologous iliac bone at L4-L5 and T10-T11.

**Fig. 6:**
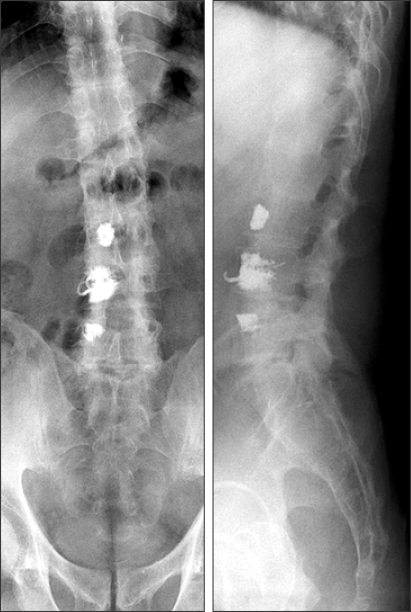
X-ray at final follow-up (2.4 years after second operation) showed interbody fusion at L4-L5 and T10-T11.

The patient was readmitted to our hospital. We discontinued antibiotic therapy and performed needle biopsies. Culture of the biopsy specimens did not lead to identification of the causative micro-organism. Because the patient had failed to respond to antibiotic therapy and the inflammation progressed, we removed the posterior implants and performed anterior curettage and interbody fusion using autologous iliac bone grafting at the L4-L5 and T10-T11 levels (Fig. 5). Regarding the postoperative histopathological tests performed using the obtained specimens, bacterial culture was negative, but signs of chronic inflammation were noted. The PCR test specific for *Mycobacterium tuberculosis* revealed positive results in two of four specimens (one from the thoracic spine and another from a lumbar spine), so we started antituberculosis drug therapy under the suspicion of tuberculous spondylitis. Three months after the last surgery, ESR and CRP went back to normal levels, and the back pain improved. Antituberculosis drug therapy was continued for 1.4 years. At 2.4 years after the last surgery, ESR and CRP were found to be normal, and the patient complained only back stiffness when walking. Plain radiography demonstrated successful interbody fusion at the final follow-up (Fig. 6).

## DISCUSSION

Infections following spinal fusion with instrumentation have been reported to occur in 2.6% to 10% of patients.^1^,^3^,4) Infections are often attributable to organisms such as *Staphylococcus aureus*, with MRSA currently recognized as one of the major pathogens.

Generally, when postoperative infection is suspected, the acquisition of specimens precedes antibiotic treatment. However, the patient in our report was imme-diately put on antibiotic therapy without specimen acquisition. Due to the inappropriate early treatments, identification of bacteria was not achievable in later biopsies. In addition, the department of anesthesiology and pain medicine carried out vertebroplasty when pain relief was not obtained with a facet joint block; this procedure had been performed to deal with severe low back pain in the absence of abnormal MRI signals. However, the employment of vertebroplasty was brash, given that such a procedure should not be utilized when ESR and CRP are high and infection is suspected.

Specimens can be obtained using needle biopsy or surgical biopsy. Needle biopsies are known to be highly successful in garnering positive cultures (68 to 96%). How-ever, some authors have reported contrary results. Using computed tomography-guided needle biopsy, Sucu et al.5) was able to obtain positive culture results from biopsy specimens in only 10 of 73 cases of inflammatory spondylitis. Hwang et al.6) also reported that the same biopsy technique permitted them to identify the causative organism in 22 (34%) of 58 patients with the same disease. In the current case, needle biopsies performed at the other two hospitals led to negative culture results and false-positive results due to contamination. In addition, although we performed tissue typing, bacterial culture testing, and PCR testing for *Mycobacterium tuberculosis* with specimens obtained using needle biopsy, the causative bacterium was not found.

Definitive diagnosis of tuberculous spondylitis is based on pathological study. Unfortunately, the culture and identification of this bacterium usually takes 6-8 weeks and has a low sensitivity of 50%. Lee et al.7) reported positive culture results in only 27 (62%) of 47 patients with tuberculous spondylitis. Against this backdrop, PCR technology has recently been recognized as a useful method for expediting diagnosis and treatment by amplifying DNA in specimens. Park et al.8) used this technology in 28 patients diagnosed clinically with tuberculosis and having pathological signs of chronic but not granulomatous inflammation. With 10 positive results in the study, the investigators concluded that PCR technology is necessary for diagnosing such patients. In the current case, we speculated that *Mycobacterium tuberculosis* was responsible for the patient's infection based on two major findings. First, although signs of chronic but not granulomatous inflammation were observed, specimens collected between the instrumentation site and the T10-T11 level had positive PCR results for *Mycobacterium tuberculosis*. Second, the patient responded well to antituberculosis therapy. Considering that positive PCR results were obtained from two biopsy sites, it appears that tuberculous spondylitis progressed from the T4-T5 level to the T10-T11 level. We attribute the failure to identify the causative micro-organism in a series of biopsies to the inherent limitations of needle biopsy and to the low identification rate of *Mycobacterium tuberculosis*.

Chang et al.9) described the differences between tuberculous spondylitis and pyogenic spondylitis. Tuber-culous spondylitis is characterized by vertebral destruction with relative preservation of the intervertebral disc and local, heterogeneous enhancement of the vertebral body. Pyogenic spondylitis is characterized by destruction of the intervertebral disc and peridiscal bone and homogeneous enhancement of the vertebral body. Jung et al.10) concluded that patients with the following MRI findings are more likely to have tuberculous spondylitis rather than pyogenic spondylitis: a well-defined, abnormal paraspinal signal; a thin, smooth abscess wall with peripheral enhancement; subligamentous extension to three or more vertebral levels; and involvement of multiple vertebral bodies and thoracic vertebrae. We also suspected the patient in our report of having tuberculous spondylitis because the early MRI scans showed signs of inflammation in several vertebral bodies along with an epidural abscess. Subligamentous extension of inflammation was seen on the follow-up MRI.

The patient in our report underwent surgery for persistent spondylitis and had positive PCR results for *Mycobacterium tuberculosis*. Based on the results of the PCR analysis performed with specimens obtained during surgery, we started antituberculosis therapy and finally observed clinical improvement. Accordingly, we believe that *Mycobacterium tuberculosis* was responsible for the patient's condition. However, it is hard to determine if the infection occurred immediately after the initial surgery or was due to superinfection. We suggest that *Mycobacterium tuberculosis* should also be taken into consideration when dealing with patients with infection following spinal surgery, if the identification of bacteria is not possible, the progress of the disease is slow, and the antibiotic treatment appears ineffective.
